# Mindfulness and Psychological Distress in College Student-Athletes: The Mediating Roles of Cognitive Reappraisal and Subjective Vitality

**DOI:** 10.3390/bs16061033

**Published:** 2026-06-20

**Authors:** Xing Liu, Li Li, Huilin Wang

**Affiliations:** 1 School of Physical Education, Hunan University of Science and Technology, Xiangtan 411201, China; 24011701010@mail.hnust.edu.cn; 2 School of Business, Hunan University of Science and Technology, Xiangtan 411201, China; 1150141@hnust.edu.cn

**Keywords:** mindfulness, cognitive reappraisal, subjective vitality, psychological distress, college student-athletes

## Abstract

Introduction: College student-athletes must often balance academic responsibilities with intensive training and competition, placing them under considerable pressure and potentially increasing their risk of mental health difficulties. Against this background, the present study focused on the link between mindfulness and psychological distress and examined whether cognitive reappraisal and subjective vitality were statistically involved in this association as indirect associations. Methods: Participants were 430 college student-athletes recruited from five universities in Hunan Province, China. Using a cross-sectional survey design, the hypothesized model was tested using structural equation modeling in AMOS 23.0, and indirect associations were examined with bootstrap analysis based on 5000 resamples. Results: Mindfulness was positively associated with both cognitive reappraisal and subjective vitality. Cognitive reappraisal was positively associated with subjective vitality but negatively associated with psychological distress. Subjective vitality also showed a negative association with distress. Moreover, mindfulness showed an indirect association with lower distress through cognitive reappraisal and subjective vitality. Discussion: The findings may contribute to a better understanding of the psychological correlates associated with mental health in college student-athletes. They also suggest that mindfulness-related psychological resources may be associated with lower distress and may help guide future longitudinal and intervention research in this group.

## 1. Introduction

College student-athletes hold dual roles as both athletes and students. They must cope with intensive training, competitive demands, and academic responsibilities at the same time, which may increase their vulnerability to physical and psychological strain (Thompson et al., 2024). Depression risk in this population ranges from 15.6% to 33.2%, and 19.1% report anxiety symptoms (Weber et al., 2023). Key stressors include balancing sport and study, uncertainty about future athletic careers, and financial pressure (Kegelaers et al., 2024). When these pressures accumulate over time, they may contribute to anxiety, depression, and emotional exhaustion (Beable et al., 2017).

These concerns are also consistent with the broader literature on university students’ mental health. A review by Roy et al. (2025) suggests that psychological distress is shaped by multiple levels of factors, including individual, interpersonal, institutional, and sociocultural influences, and has become a prominent mental health issue among Chinese university students (Lin et al., 2025). Compared with other university students, college student-athletes devote substantial time and energy to training, competition, and team-related activities, which may further intensify sport–academic role conflict and psychological burden, with potential implications for academic achievement, athletic performance, and overall well-being (Lopes Dos Santos et al., 2020; O’Neil et al., 2021). In addition, athletic identity, gender differences, and coach–athlete relationships are all associated with athletes’ psychological adjustment (Stokowski et al., 2022; Kew et al., 2024; Kernan et al., 2025). In the context of Chinese collegiate competitive sport, the dual demands of sport and study are also embedded within the institutional arrangements surrounding student-athletes and may be reinforced by collectivistic cultural expectations (Zhang & Khan, 2025; Song et al., 2024). Moreover, collectivistic orientation, coach support, emotion regulation, and cognitive reappraisal are closely related to sport-related adjustment (Li et al., 2024).

Mindfulness is generally conceptualized as intentionally directing attention to ongoing experience while maintaining an orientation of openness, acceptance, and non-judgment, and it may be treated either as a momentary state of consciousness or as a relatively enduring psychological tendency (Bishop et al., 2004). Evidence from both general populations and sport-related research suggests that higher levels of mindfulness are associated with more adaptive emotion regulation, lower psychological stress, and better psychological functioning (Keng et al., 2011; Khoury et al., 2013). Recent reviews and meta-analytic evidence further indicate that mindfulness-based interventions are associated with lower levels of anxiety, depressive symptoms, and psychological distress among student populations (Xu et al., 2025; da Silva et al., 2023). In sport contexts, mindfulness has also been linked to fewer symptoms of anxiety, depression, and stress, as well as higher levels of well-being (Mohammed et al., 2018; Lee et al., 2024; Zheng et al., 2022). For college student-athletes, this construct may be particularly relevant, as present-moment, non-judgmental awareness may be closely related to how athletes cope with the combined pressures of training, competition, and academic life (Coffey & Hartman, 2008; Yuan et al., 2025).

Although previous research has examined mindfulness among general university students and broader athlete populations (Noetel et al., 2019; Johnson et al., 2023), the association between mindfulness and psychological distress among college student-athletes remains insufficiently clarified, particularly within the context of Chinese collegiate competitive sport. Existing studies have mainly explained the link between mindfulness and mental health through stress appraisal, coping styles, and broad emotion regulation processes (McBride et al., 2022; O’Connor et al., 2023; Maddock & Blair, 2023). However, for Chinese college student-athletes, limited research has jointly considered appraisal-related regulation processes and energy-related positive psychological resources. In this context, cognitive reappraisal and subjective vitality may be especially important. The former reflects athletes’ flexible reinterpretation of stressful experiences, whereas the latter reflects their perceived psychological energy, which may be necessary for sustaining engagement under continuous academic and athletic demands. Therefore, the present study aims to clarify how mindfulness is associated with psychological distress through stress reinterpretation and psychological energy within the culturally and institutionally specific context of Chinese collegiate sport.

Based on this theoretical logic, mindfulness may be associated with psychological distress not only directly, but also through regulation-related and vitality-related processes. Cognitive reappraisal may represent an appraisal-related process, reflecting how mindful awareness is associated with more flexible interpretations of stressful experiences. Subjective vitality may represent an energy-related resource that supports psychological adjustment under sustained sport–academic demands (Buhle et al., 2014; Ryan & Deci, 2008). On this basis, the present study pursued three objectives: first, to explore the associations among mindfulness, cognitive reappraisal, subjective vitality, and psychological distress in college student-athletes; second, to examine the indirect association between mindfulness and psychological distress through cognitive reappraisal and subjective vitality; and third, to examine whether cognitive reappraisal and subjective vitality can be integrated into a sequential indirect association between mindfulness and psychological distress.

## 2. Literature Review and Research Hypotheses

### 2.1. Conceptual Definitions

#### 2.1.1. Mindfulness

In the present study, mindfulness is treated as a measurable self-regulatory tendency characterized by an individual’s ability to attend to present-moment internal and external experiences in an accepting manner, rather than primarily as a philosophical concept (Kabat-Zinn, 2003; Brown & Ryan, 2003). Among college student-athletes, mindfulness can be understood as a relatively stable tendency to maintain awareness of task demands, internal states, and bodily responses during academic and athletic activities, while approaching these experiences with acceptance, non-judgment, and less automatic reactivity. Research in sport psychology has examined mindfulness and acceptance-based approaches in relation to present-moment focus, attentional control, stress coping, emotion regulation, and adaptive responses to performance-related setbacks (Bernier et al., 2009; Sappington & Longshore, 2015; Bühlmayer et al., 2017; Noetel et al., 2019). More specifically, studies involving student-athletes have linked mindfulness to life stress, coping efficacy, and decision-related rumination, suggesting that mindfulness is particularly relevant for understanding how this population manages the dual demands of academic and athletic life (Kaiseler et al., 2017).

#### 2.1.2. Cognitive Reappraisal

Cognitive reappraisal is a key process for emotional adjustment and mental health outcomes (McRae, 2016). Cognitive appraisal theory holds that emotional responses depend not simply on situations themselves, but on how individuals interpret them (Lazarus & Alfert, 1964; Lazarus, 1966). From this appraisal-based perspective, cognitive reappraisal is commonly understood as an antecedent-focused emotion regulation strategy, whereby individuals reinterpret emotion-eliciting situations before emotional responses are fully generated (Gross, 1998).

For college student-athletes, cognitive reappraisal is an important regulatory strategy for managing stress-related emotions and maintaining psychological adjustment (Cui et al., 2024). In sport contexts, a growing body of research has examined cognitive reappraisal as part of athletes’ emotion regulation and adaptive responses to performance-related demands (Robazza et al., 2023; Kim & Tamminen, 2023). In the Chinese sport context, cognitive reappraisal, collectivistic orientation, and coach support have also been closely linked to sport-related adjustment (Li et al., 2024). When setbacks, high training loads, or within-team competition are appraised as threats to competence or self-worth, they may be accompanied by stronger negative emotions (Robazza et al., 2023). Cognitive reappraisal reflects a more flexible interpretation of such experiences, for example, viewing competitive setbacks or intensive training as information for improvement rather than as evidence of failure.

#### 2.1.3. Subjective Vitality

In positive and health psychology, subjective vitality is commonly used to describe the conscious experience of energy and aliveness (Frederick & Ryan, 2023). Ryan and Frederick (1997) argued that it represents more than physical stamina or temporary excitement, instead reflecting a positive state rooted in the activation of inner psychological resources. Conceptually, subjective vitality is distinct from broader constructs such as general well-being, engagement, recovery, and motivation. More specifically, it captures individuals’ subjective experience of feeling energetic and alive. Because college student-athletes are required to sustain psychological energy while simultaneously facing academic and athletic demands, subjective vitality primarily reflects the energy-related dimension of positive functioning rather than a broad indicator of well-being. Evidence from sport, education, and physical education populations suggests that positive energy is associated with engagement, adjustment, autonomous motivation, and academic functioning, further supporting the importance of examining vitality-related resources in physical activity-related educational contexts (Kinnafick et al., 2014; Adie et al., 2008; Fini et al., 2010; Zach & Inglis, 2019).

For college student-athletes, psychological resource depletion may become more evident, leading to emotional fluctuation, exhaustion, and adjustment difficulties (Parker et al., 2021). As a positive psychological resource, subjective vitality has been associated with healthier functioning, persistence, recovery, and adaptive capacity under sport-related demands (Wang et al., 2023; Chang et al., 2018). Therefore, in the present study, subjective vitality is conceptualized as college student-athletes’ perceived sense of energy and aliveness when facing multiple stressors.

#### 2.1.4. Psychological Distress

In public health, epidemiological studies, and clinical intervention research, psychological distress is frequently regarded as an important indicator of mental health status (Drapeau et al., 2012). Rather than referring to a specific psychiatric disorder, it is used to describe a spectrum of adverse mental experiences, including anxiety, depression, irritability, helplessness, tension, and emotional exhaustion, often together with impaired psychological functioning (Folorunsho et al., 2025; Veit & Ware, 1983). In the present study, psychological distress is conceptualized as a relatively persistent negative psychological state experienced under sustained sport–academic demands.

For college student-athletes, psychological distress is better understood as a broader adjustment-related state rather than a transient emotional reaction, as it often emerges alongside continuous and overlapping demands related to training, competition, academic work, injury recovery, role conflict, and career development (Bissett & Tamminen, 2022; McCluskey et al., 2025; Kegelaers et al., 2024). Research on athlete mental health has linked higher levels of psychological distress to poorer well-being, recovery, engagement, and academic or sport-related adjustment, suggesting that psychological distress is an important outcome variable for understanding student-athletes’ functioning under multiple demands (Arnold & Fletcher, 2012; Liu et al., 2022; Muhammad et al., 2021).

### 2.2. Hypotheses

#### 2.2.1. Mindfulness, Cognitive Reappraisal, and Subjective Vitality

Success in competitive sport is shaped not only by physical and technical capacities, but also by the ability to regulate oneself effectively under pressure (Kirschenbaum, 1987). Among college student-athletes, performance is influenced in part by how stressors such as worry, tension, and negative self-evaluation are interpreted and managed (Jones et al., 2009; Zhong et al., 2025). When these pressures continue over time, psychological resources may be worn down, making positive functioning increasingly difficult to maintain (Löw et al., 2020). Mindfulness cannot eliminate such external demands, but it may be associated with less overinvolvement in negative emotional experiences by encouraging a more open and non-evaluative awareness of present experience (Goyal et al., 2014; Lindsay & Creswell, 2017).

Mindfulness and cognitive reappraisal are both considered adaptive emotion regulation resources, but they reflect different regulatory processes (Chambers et al., 2009). Reappraisal involves reconstructing the meaning of a situation (Ochsner & Gross, 2005), whereas mindfulness emphasizes awareness and acceptance of present experience (Kabat-Zinn, 2003). Rather than directly changing thought content, mindfulness may first be related to decentering from automatic reactions, which may be relevant to the flexible reinterpretation of stressful experiences. Consistent with this view, mindfulness has been linked to decentering, cognitive flexibility, and greater use of reappraisal, both in general contexts and in sport settings (E. Garland et al., 2009; Zhou et al., 2023; Robazza et al., 2024).

A similar pattern appears in relation to subjective vitality. In addition to its connections with mental health and well-being (Keng et al., 2011), mindfulness has also been related to the maintenance of positive psychological energy and adaptive functioning (Chang et al., 2018; Chang et al., 2022; Brown & Ryan, 2003). Reappraisal has likewise been associated with positive affect, well-being, and adaptive functioning, all of which are closely linked to subjective vitality (Gross & John, 2003; Hu et al., 2014). Findings from sport research further suggest that reappraisal may preserve positive affect and support pleasant emotional experiences under daily stress(Doorley & Kashdan, 2021; Robazza et al., 2023). Together, this evidence provides a basis for expecting positive associations among mindfulness, cognitive reappraisal, and subjective vitality. Accordingly, the following hypotheses are proposed:

**Hypothesis** **1 (H1).** *Mindfulness is positively associated with cognitive reappraisal among college student-athletes.*

**Hypothesis** **2 (H2).** *Mindfulness is positively associated with subjective vitality among college student-athletes.*

**Hypothesis** **3 (H3).** *Cognitive reappraisal is positively associated with subjective vitality among college student-athletes.*

#### 2.2.2. Cognitive Reappraisal, Subjective Vitality, and Psychological Distress

As an antecedent-focused regulatory process, cognitive reappraisal helps individuals influence emotional outcomes by changing how emotion-eliciting situations are interpreted before emotional responses become fully activated (Gross, 2001). This distinguishes it from response-focused strategies such as suppression, which take place after emotions have already arisen and are generally viewed as less adaptive (Aldao et al., 2010). Because emotional responses depend partly on how situations are interpreted, more constructive appraisals of stressful or threatening events may reduce perceived threat and thereby lower anxiety, depressive symptoms, and tension (Webb et al., 2012; Riepenhausen et al., 2022). Empirical findings further indicate that individuals with more frequent use of reappraisal are likely to report fewer negative emotions, better well-being, and lower levels of depressive symptoms (Gross & John, 2003; Eftekhari et al., 2009). These findings imply that reappraisal may reduce psychological distress by reshaping the meaning of adverse experiences and supporting recovery from them.

Subjective vitality reflects the psychological energy available to individuals during adaptive functioning (Ryan & Deci, 2008). Higher vitality has been linked to stronger control, greater psychological resilience, and lower levels of anxiety, depression, and stress, partly because it helps individuals stay engaged in demanding situations and recover more quickly from emotional depletion (Bostic et al., 2000; Kaya, 2024). It has also been viewed as a beneficial psychological resource that can buffer the harmful effects of stress and reduce distress (Mansell & Turner, 2023). Following this line of reasoning, the present study proposes the following hypotheses:

**Hypothesis** **4 (H4).** *Cognitive reappraisal is negatively associated with psychological distress among college student-athletes.*

**Hypothesis** **5 (H5).** *Subjective vitality is negatively associated with psychological distress among college student-athletes.*

#### 2.2.3. Mediating Effect

Cognitive reappraisal and subjective vitality may represent two important pathways through which mindfulness relates to psychological distress. Reappraisal allows stressful experiences to be interpreted in a more adaptive manner, which can reduce negative emotional responses and facilitate better adjustment (Stover et al., 2024). Subjective vitality reflects positive psychological energy and functioning and is associated with lower exhaustion (Frederick & Ryan, 2023; E. L. Garland et al., 2015). From this perspective, mindfulness may reduce automatic reactivity to stress by fostering greater awareness and non-judgment toward present experience, while also making adaptive reappraisal more likely (Chambers et al., 2009). Because cognitive reappraisal involves how individuals cognitively organize stressful experiences, it may represent a regulation process that is more proximal to mindful awareness and may link mindful awareness to vitality-related functioning (Maras & Leger, 2024). Higher subjective vitality may then further support engagement, perceived control, and recovery, thereby helping to reduce anxiety, depression, and tension (Muraven et al., 2008). Therefore, cognitive reappraisal and subjective vitality may not only operate as independent mediators, but may also form a sequential process in which cognitive reappraisal precedes subjective vitality in the association between mindfulness and psychological distress. Following this logic, the present study proposes the following hypothesis:

**Hypothesis** **6 (H6).** *The association between mindfulness and psychological distress is statistically indirect through cognitive reappraisal and subjective vitality among college student-athletes.*

**Figure 1:**
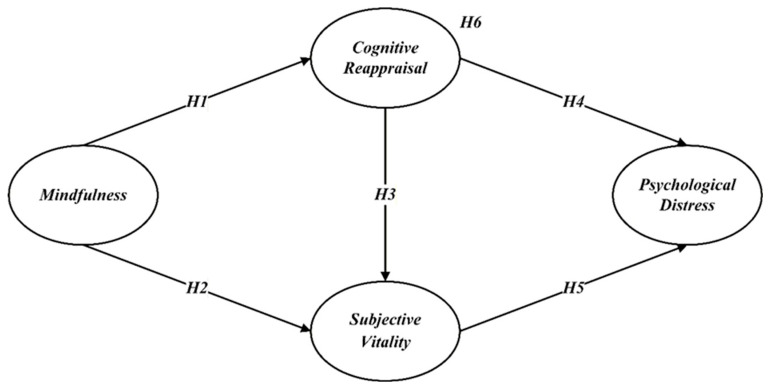
Hypothesized Model. Note: Because the present study focused on the sequential indirect pathway through cognitive reappraisal and subjective vitality, the direct path from mindfulness to psychological distress was omitted and not estimated.

The proposed hypotheses are summarized in Figure 1. The arrows indicate theoretically hypothesized directions; however, because the study used a cross-sectional design, the model does not establish temporal or causal ordering among the variables. Moreover, the present data cannot demonstrate that this ordering is superior to other possible arrangements of the variables.

## 3. Methods

### 3.1. Participants and Procedures

The study was carried out in December 2025 across five universities in Hunan Province, China. Given that the present study focused on college student-athletes, participants were understood as university students who reported a primary sport event and regular sport training experience. This definition was based on self-reported sport involvement rather than a single formal registration or team membership criterion. Therefore, the sample may include students with heterogeneous levels of athletic training and competitive involvement. Their sport involvement and competitive experience were further described using self-reported information on sport type, weekly training frequency, average training duration, and participation in formal competitions during the previous 12 months.

A snowball recruitment strategy was used through the Schools of Physical Education. Although this approach was useful for reaching college student-athletes across different universities, it may have introduced selection bias and should therefore be acknowledged as a sampling limitation. At each institution, the research team first approached teachers and student leaders, who assisted in identifying and inviting eligible college student-athletes. Those initial respondents were then encouraged to pass the paper questionnaire to other qualified student-athletes within their own networks. In total, 500 questionnaires were circulated. Of these, 468 were returned, corresponding to a response rate of 93.60%. After excluding cases with missing data or invariant response patterns, 430 usable questionnaires remained and were included in the final analysis, yielding an effective response rate of 86.00%.

In this way, the sample was characterized according to participants’ self-reported athletic background rather than by a single formal membership criterion. Specifically, the questionnaire recorded their primary sport event, average weekly training frequency, average duration of each training session, and whether they had participated in formal competitions during the previous 12 months. These variables allowed the study to clarify the nature of participants’ sport participation and to distinguish their level of training and competitive experience within the college student-athlete sample. Accordingly, the findings should be interpreted as reflecting this specific sample of college students with sport training experience, rather than as representative of all college student-athletes in China.

Table 1 shows the characteristics of the final sample. Among the 430 participants, 55.1% were men and 44.9% were women. The largest age groups were 20–21 years (37.4%) and 22–23 years (29.8%). Team ball sports (30.5%) and track and field (25.8%) accounted for the largest proportions of sport type. It should be noted that these sport categories were used to describe the overall sample composition and may include sports with different physical demands, training loads, and competitive characteristics. Most participants reported training three to four times per week (44.2%), with a typical session lasting two to three hours (54.2%). In addition, 84.0% had taken part in formal competitions during the previous 12 months, whereas 16.0% had not, further indicating variation in competitive involvement within the sample.

### 3.2. Instruments

The survey included two components: a demographic section and the core study measures. Demographic information covered gender, age, primary sport event, average weekly training frequency, typical duration of each training session, and whether participants had taken part in formal competitions during the previous 12 months.

All core measures were administered in Chinese. As formally validated Chinese versions specifically for college student-athletes were not available for all scales, the English items were translated into Chinese following a translation and back-translation procedure. Two bilingual researchers first translated the items into Chinese, and another bilingual researcher back-translated them into English. Discrepancies were discussed and resolved to ensure semantic consistency with the original items. Given that these constructs were originally developed and validated in different populations and cultural contexts, we did not assume full equivalence in meaning or measurement properties. Instead, the translated items were reviewed to ensure that the meanings of mindfulness, cognitive reappraisal, subjective vitality, and psychological distress were understandable and appropriate within the Chinese college student-athlete context. The psychometric properties of the Chinese measures were further examined in the present sample, and the results should be interpreted as evidence of acceptable measurement performance in this sample rather than as full validation of these measures in the broader Chinese college student-athlete population.

Mindfulness was assessed with the 5-item Mindful Attention Awareness Scale (MAAS-5) (Van Dam et al., 2010). Responses were recorded on a 5-point Likert scale ranging from 1 (strongly disagree) to 5 (strongly agree). A sample item is “I find myself doing things without paying attention.” Because all items were negatively worded, they were reverse-coded prior to analysis so that higher scores indicated higher levels of mindfulness.

Cognitive reappraisal was captured using the 6-item cognitive reappraisal dimension of the Emotion Regulation Questionnaire–Short Form (ERQ-S) (Preece et al., 2023). Responses were given on the same 5-point response format, from 1 (strongly disagree) to 5 (strongly agree), with higher values indicating greater use of reappraisal strategies. A sample item is “When I want to feel less negative emotion, I change the way I’m thinking about the situation.”

Subjective vitality was measured with a 6-item revised form of the Subjective Vitality Scale, originally proposed by Ryan and Frederick (1997) and later validated by Bostic et al. (2000). Participants responded on a 5-point scale from 1 (strongly disagree) to 5 (strongly agree), and higher scores indicated stronger subjective vitality. A representative item is “I feel alive and vital.”

Psychological distress was evaluated with the 6-item Kessler Psychological Distress Scale (K6) (Kessler et al., 2002). Participants reported how often they had experienced each feeling during the past 30 days using a 5-point response scale from 1 (never) to 5 (always). Higher scores reflected greater psychological distress. A sample item is “During the last 30 days, about how often did you feel hopeless?”

### 3.3. Data Analysis

All data analyses were performed using IBM SPSS 26.0 and AMOS 23.0. Before conducting SEM, the data were screened for missing values, invariant response patterns, and the distributional properties of the observed variables. Cases with missing data or invariant response patterns were excluded from the analysis. The SEM analyses were conducted using maximum likelihood estimation. Descriptive statistics together with Pearson correlation coefficients were first generated in SPSS, and AMOS was then used to conduct confirmatory factor analysis (CFA) on the measurement model. In line with the two-step SEM approach, the adequacy of the measurement model was evaluated prior to estimation of the structural model. Reliability and validity were evaluated for each construct through internal consistency, convergent validity, and discriminant validity. The structural model was then applied to examine the proposed associations among mindfulness, cognitive reappraisal, subjective vitality, and psychological distress. Model adequacy was judged on the basis of multiple fit indices, and standardized path coefficients were reported. To evaluate indirect effects, bootstrap procedures with 5000 resamples and 95% bias-corrected confidence intervals were employed.

Possible common method variance (CMV) was examined by contrasting a single-factor solution with the proposed four-factor measurement model. The one-factor model showed a substantially poorer fit to the data, χ^2^ = 4601.456, df = 252, *p* < 0.001, whereas the four-factor model demonstrated a much better fit, χ^2^ = 284.837, df = 224, *p* < 0.001. The results suggest that CMV is unlikely to be a serious threat to the findings. However, given the cross-sectional self-report design, the possibility of common method bias cannot be completely ruled out.

## 4. Results

### 4.1. Measurement Model

The latent constructs were evaluated using confirmatory factor analysis in AMOS 23.0. As reported in Table 2, Cronbach’s α and composite reliability (CR) both fell between 0.876 and 0.898, indicating strong internal consistency. Average variance extracted (AVE) values ranged from 0.585 to 0.596, all exceeding the recommended cutoff of 0.50, which supports convergent validity. Standardized factor loadings were acceptable across all constructs, ranging from 0.738 to 0.801 for Subjective Vitality, 0.748 to 0.779 for Mindfulness, 0.764 to 0.778 for Cognitive Reappraisal, and 0.738 to 0.785 for Psychological Distress.

Evidence for discriminant validity is presented in Table 3. The square roots of the AVE estimates ranged between 0.765 and 0.772 and were greater than the corresponding inter-construct correlations, indicating that the four constructs were empirically distinguishable.

### 4.2. Structural Model

Once the measurement model had been established, the hypothesized structural relationships were estimated in AMOS 23.0. The model showed a satisfactory fit to the data, with χ^2^ = 292.702, df = 225, χ^2^/df = 1.301, RMR = 0.047, GFI = 0.943, AGFI = 0.930, IFI = 0.948, TLI = 0.987, and CFI = 0.986.

**Figure 2:**
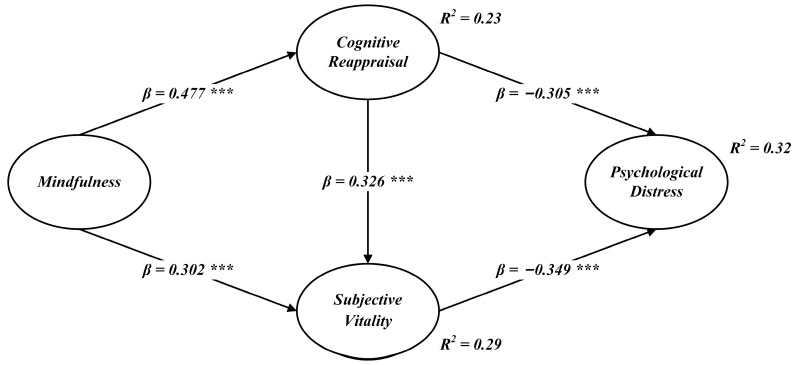
Structural path model. Note: *** *p* < 0.001. The total indirect effect from mindfulness to psychological distress was −0.206, 95% Boot CI [−0.261, −0.153].

As shown in Figure 2, mindfulness exhibited positive associations with cognitive reappraisal (β = 0.477, *p* < 0.001) and subjective vitality (β = 0.302, *p* < 0.001), supporting H1 and H2. Cognitive reappraisal was likewise positively related to subjective vitality (β = 0.326, *p* < 0.001), supporting H3. In addition, cognitive reappraisal showed a negative association with psychological distress (β = −0.305, *p* < 0.001), and subjective vitality was similarly negatively linked to psychological distress (β = −0.349, *p* < 0.001), supporting H4 and H5. In terms of effect magnitude, these standardized coefficients indicate moderate associations, with the strongest path observed from mindfulness to cognitive reappraisal. This suggests that mindfulness may have practical relevance for college student-athletes’ ability to reinterpret stressful experiences, while cognitive reappraisal and subjective vitality may be meaningfully associated with lower psychological distress. In terms of explained variance, the model accounted for 23% of cognitive reappraisal, 29% of subjective vitality, and 32% of psychological distress.

Bootstrap procedures based on 5000 resamples were used to examine the indirect effects (see Table 4). The total indirect effect of mindfulness on psychological distress was significant (indirect effect = −0.206, Boot SE = 0.028, 95% Boot CI [−0.261, −0.153]). In addition, the specific indirect effect through cognitive reappraisal was significant (indirect effect = −0.097, Boot SE = 0.022, 95% Boot CI [−0.142, −0.055]), as was the specific indirect effect through subjective vitality (indirect effect = −0.109, Boot SE = 0.021, 95% Boot CI [−0.150, −0.070]). Because the confidence intervals did not include zero, these results are consistent with H6 in terms of statistical indirect associations.

## 5. Discussion

### 5.1. Theoretical Contributions

The present study extends research on the mental health of college student-athletes by exploring the interrelationships among mindfulness, cognitive reappraisal, subjective vitality, and psychological distress, as well as the indirect associations among these variables. Overall, the findings point to mindfulness as an important psychological correlate and suggest that mindfulness showed a statistically significant indirect association with lower distress when cognitive reappraisal and subjective vitality were included in the model. More specifically, the contribution of this study lies in situating these associations within the context of Chinese collegiate competitive sport. In this context, student-athletes’ psychological adjustment may be shaped not only by the dual demands of sport and study, but also by cultural expectations related to regulation, persistence, and collective adaptation. At the same time, the explained variance values for cognitive reappraisal, subjective vitality, and psychological distress were moderate, indicating that a substantial proportion of variance remains unexplained. Therefore, the present model should be understood as a partial explanation of athlete distress rather than a comprehensive account. Other contextual and interpersonal factors, such as coach support, team climate, academic stress, training load, injury experience, and social support, may also contribute to psychological distress among college student-athletes.

First, the findings help clarify the pattern of associations among the four variables. Higher mindfulness was accompanied by higher cognitive reappraisal and greater subjective vitality, while both cognitive reappraisal and subjective vitality were associated with lower psychological distress. Cognitive reappraisal was likewise positively connected with subjective vitality. These findings are broadly consistent with earlier evidence (Theodorou et al., 2023; Zhou et al., 2023; Chang et al., 2022) and indicate that, in college student-athletes, mindfulness should not be understood only as awareness of present experience. Rather, it may also represent a broader psychological resource associated with more adaptive regulation and better functioning. More importantly, these associations suggest that the role of mindfulness in this population may be embedded within a broader regulatory context. College student-athletes who report higher levels of mindfulness may be more likely to engage in adaptive cognitive appraisal and may also maintain a stronger sense of psychological energy. In the context of Chinese collegiate competitive sport, athletes are often required to continuously regulate themselves across training discipline, athletic performance, academic responsibilities, and team expectations. In this sense, mindfulness may function not only as a quality of attention, but also as a psychological characteristic closely related to emotion regulation and positive functioning in both sport and academic contexts.

Second, the sequential indirect association provides additional evidence regarding the statistical association between mindfulness and psychological distress. The relationship between mindfulness and psychological distress was reflected not only in their direct association, but also in the theoretically informed ordering of cognitive reappraisal and subjective vitality. This interpretation is consistent with previous research linking mindfulness to cognitive regulation and psychological functioning (Brown & Ryan, 2003; Theodorou et al., 2023). More specifically, the findings suggest that the association between mindfulness and psychological distress may be partly understood through appraisal-related and vitality-related characteristics. In contexts involving intensive training, competitive setbacks, or academic pressure, higher mindfulness among college student-athletes may be associated with a stronger tendency to flexibly reinterpret stressful events, as well as with higher levels of subjective vitality. Together, these characteristics may form an adaptive psychological profile associated with lower psychological distress, although the cross-sectional design does not allow the temporal ordering of these variables to be determined (Hill & Updegraff, 2011; Lindsay & Creswell, 2017). Therefore, the sequential pathway proposed in this study should be understood as a theoretically informed explanatory framework rather than as a process whose temporal sequence has been empirically established. Alternative model specifications may fit the data equally well.

Finally, these findings extend our understanding of psychological adjustment among college student-athletes by highlighting the joint importance of regulatory resources and vitality-related resources. Focusing only on exposure to sport, academic, or interpersonal stressors may be insufficient for fully understanding psychological distress in this population. It is also necessary to consider how athletes appraise stressful experiences and whether they are able to maintain sufficient psychological energy to remain engaged in demanding sport–academic environments. In this context, cognitive reappraisal represents an adaptive appraisal-related characteristic, whereas subjective vitality reflects a positive psychological resource that may accompany better adjustment. This interpretation accords with earlier work indicating that positive regulatory resources and subjective vitality are closely related to psychological adjustment (Ryan & Deci, 2008; Wang et al., 2023). Therefore, mental health promotion for college student-athletes should consider not only the reduction in problems, but also the potential associations among mindfulness, cognitive reappraisal, and subjective vitality.

### 5.2. Practical Implications

The findings suggest that mindfulness is related to college student-athletes’ psychological adjustment in training, competition, academic, and coach-related contexts. Given the cross-sectional design of this study, the following implications should be interpreted not as direct practical recommendations, but as possible future intervention targets for applied research.

At the university level, mental health promotion can be considered as part of a broader athlete development system (Kegelaers et al., 2024). Future intervention studies could examine whether university-based support targeting present-moment awareness, cognitive regulation, and psychological services contributes to better psychological adjustment among student-athletes.

At the coach level, coach education programs may consider mindfulness cultivation, cognitive reappraisal guidance, and the maintenance of subjective vitality as potential intervention targets rather than established practices. Future programs could further examine whether supportive communication, constructive feedback, and process-oriented evaluation help create a training environment that enables athletes to reinterpret setbacks and maintain psychological vitality (Robazza et al., 2022). At the same time, training load and psychological pressure also deserve attention (Bonin et al., 2025).

At the individual athlete level, student-athletes may consider mindfulness, awareness of stressful experiences, and flexible interpretations of setbacks or role conflict as potential self-regulatory resources (Sappington & Longshore, 2015). They may also benefit from paying attention to rest, recovery, and personal interests, as these factors may be related to subjective vitality. Overall, mindfulness, cognitive reappraisal, and subjective vitality may serve as promising targets for future intervention research aimed at supporting psychological adjustment among college student-athletes.

### 5.3. Limitations

This research has several limitations. First, the cross-sectional design restricts causal interpretation and does not allow examination of temporal ordering or psychological change over time. Because all variables were measured at a single time point, the study cannot determine whether mindfulness, cognitive reappraisal, subjective vitality, and psychological distress change across different training, competition, or academic periods. Future studies should use longitudinal or intervention-based designs to examine these dynamic processes more clearly.

Second, all variables were measured using self-report questionnaires, which may increase the risk of common method bias and social desirability bias. Participants may have underreported psychological distress or overreported positive psychological resources. Although a single-factor test was conducted as a basic diagnostic check, this procedure cannot fully rule out common method variance. Future research should consider multi-source data, repeated measurements, or behavioral and clinical indicators where appropriate.

Third, the use of snowball sampling may have introduced selection bias and limited sample representativeness. Because recruitment was assisted by teachers and student leaders, students who were more connected to these networks may have been more likely to participate, whereas those experiencing greater stress, fatigue, injury, or distress may have been less willing or available to respond. Future studies should adopt more systematic sampling strategies and include student-athletes from a wider range of universities and regions.

Fourth, participants were recruited only from universities in Hunan Province, China. Therefore, the findings may reflect specific cultural and educational characteristics of Chinese college student-athletes, including how mindfulness, emotional expression, distress, and help-seeking are understood and reported. Future research should examine whether the present model applies to other cultural and institutional contexts.

Finally, the study did not fully capture sport-specific differences. Although sport type, training frequency, training duration, and competition participation were collected, these variables were mainly used to describe the sample rather than being fully incorporated into the structural model. In addition, athlete-specific stressors, such as academic–sport role conflict, training pressure, injury experience, coach-related pressure, selection anxiety, and competition demands, were not directly measured. The broad sport categories used in this study may also include sports with different physical demands and competitive characteristics. Future studies should use more fine-grained sport classifications and include contextual sport-specific variables to provide a more athlete-focused understanding of psychological distress.

## 6. Conclusions

This study suggests that psychological distress among college student-athletes is meaningfully related to mindfulness, cognitive reappraisal, and subjective vitality. Higher levels of mindfulness were linked to greater cognitive reappraisal, greater subjective vitality, and lower distress. When cognitive reappraisal and subjective vitality were included in the model, mindfulness showed a statistically significant indirect association with psychological distress, suggesting that this relationship may involve multiple interconnected psychological characteristics.

Taken together, these findings contribute to understanding factors associated with psychological distress in college student-athletes and provide cautious implications for future applied work. Mindfulness, cognitive reappraisal, and subjective vitality may serve as useful reference points for future efforts to support student-athletes’ psychological adjustment. When psychological distress is evident or persistent, timely professional support may remain important for student-athletes’ long-term academic and athletic development.

## Figures and Tables

**Table 1:** Demographic characteristics (N = 430).

Variable	Category	Frequency	%
Gender	Male	237	55.1
	Female	193	44.9
Age	18–19	94	21.9
	20–21	161	37.4
	22–23	128	29.8
	24 and above	47	10.9
Primary Sport Event	Team ball sports (e.g., basketball, football, volleyball)	131	30.5
	Individual ball sports (e.g., table tennis, tennis, badminton)	102	23.7
	Track and field	111	25.8
	Combat/skill-based sports (e.g., taekwondo, martial arts, gymnastics)	64	14.9
	Other	22	5.1
Average Weekly Training Frequency	1–2 times	80	18.6
	3–4 times	190	44.2
	5–6 times	115	26.7
	7 times or above	45	10.5
Average Duration of Each Training Session	Less than 2 h	124	28.8
	2–3 h	233	54.2
	More than 3 h	73	17.0
Participation in Formal Competitions in the Past 12 Months	Yes	361	84.0
	No	69	16.0

**Table 2:** Reliability and validity.

Items	Factor Loadings	Cronbach’s α	CR	AVE
Mindfulness (MI)		0.876	0.876	0.585
MI1	0.767			
MI2	0.770			
MI3	0.760			
MI4	0.748			
MI5	0.779			
Cognitive Reappraisal (CogReap)		0.898	0.898	0.594
CogReap1	0.768			
CogReap2	0.765			
CogReap3	0.778			
CogReap4	0.772			
CogReap5	0.778			
CogReap6	0.764			
Subjective Vitality (SV)		0.898	0.898	0.596
SV1	0.738			
SV2	0.771			
SV3	0.799			
SV4	0.777			
SV5	0.801			
SV6	0.744			
Psychological Distress (PD)		0.894	0.894	0.585
PD1	0.780			
PD2	0.755			
PD3	0.785			
PD4	0.752			
PD5	0.738			
PD6	0.777			

Note: MI = Mindfulness; CogReap = Cognitive Reappraisal; SV = Subjective Vitality; PD = Psychological Distress; AVE = average variance extracted; composite reliability is reported in the CR column.

**Table 3:** Pearson correlation.

Construct	MI	CogReap	SV	PD
MI	(0.765)			
CogReap	0.420 **	(0.771)		
SV	0.402 **	0.420 **	(0.772)	
PD	−0.365 **	−0.417 **	−0.438 **	(0.765)

Note: Diagonal entries report the square roots of the AVE values, whereas the off-diagonal entries represent Pearson correlations among the constructs. ** indicates *p* < 0.01.

**Table 4:** Indirect effect.

Path	Indirect Effect	Boot SE	95% Boot CI
MI → CogReap → PD	−0.097	0.022	[−0.142, −0.055]
MI → SV → PD	−0.109	0.021	[−0.150, −0.070]
Total indirect effect: MI → PD	−0.206	0.028	[−0.261, −0.153]

## Data Availability

The data used to support the findings of this study are available from the corresponding author upon request.
